# Death of Dr. Wimberly

**Published:** 1908-06

**Authors:** 


### DEATH OF DR. WIMBERLY.

Particularly sad are the details of the death of Dr. Warren Wimberly who was nearly drowned six miles south of Jeffersonville, April 26. While on horseback on his way to visit a patient he tried to ford a stream much swollen by the recent rains. After a desperate struggle he finally reached the opposite bank, but after considerable exposure died while being taken home by some friends in a buggy. Dr. Wimberly was well known in Macon and was related to many of Georgia's well known citizens.

---

A committee has been appointed by the Wisconsin State Medical Society for the protection of its members against malpractice suits. The purpose is to obtain necessary funds, and to employ the best legal talent for the defense of any member of the society needing such services.

